# Retrospective analysis of dissemination of the 2.MED1 phylogenetic branch of *Yersinia pestis* in the Caucasus

**DOI:** 10.1371/journal.pone.0283670

**Published:** 2023-03-29

**Authors:** Galina A. Eroshenko, Alina N. Balykova, Konstantin A. Nikiforov, Yaroslav M. Krasnov, Lyubov M. Kukleva, Ekaterina A. Naryshkina, Alexander A. Kuznetsov, Nikolay V. Popov, Vladimir V. Kutyrev

**Affiliations:** Russian Research Anti-Plague Institute “Microbe”, Federal Service for Surveillance in the Sphere of Consumers Rights Protection and Human Welfare, Saratov, Russian Federation; Health Directorate, LUXEMBOURG

## Abstract

The 2.MED1 phylogenetic branch of *Yersinia pestis* of the medieval biovar became widespread in the Caspian Sea region, the Caucasus, and the Northern Aral Sea region in the 20th century, causing outbreaks and epizootics of plague there. Some of the formed natural foci of 2.MED1 still show epizootic activity and retain their epidemic potential. In this work, we carried out a phylogenetic analysis of 46 *Y*. *pestis* strains of the medieval biovar isolated in the Caucasus, the Caspian Sea, and the Northern Aral Sea regions during epidemic outbreaks and epizootics from 1922–2014. The obtained phylogenetic data, together with epidemiological and epizootological data accumulated over a period of about a hundred years, indicate the presence of two waves of penetration of the 2.MED1 branch into the Caucasus. The first occurred, apparently, in the first half of the 20th century as a result of the penetration of 2.MED1 from the foci of the Northern and North-Western Caspian Sea. The second wave was caused by the spread of 2.MED1 from the Northern Aral to the foci of the North-Western, Northern and Eastern Caspian Sea regions at the beginning of the second half of the 20th century, followed by introduction into the Pre-Caucasus and Transcaucasia. The rapid spread of 2.MED1 could be associated with the transfer of the pathogen by land and sea transport in the process of economic activity of the population.

## Introduction

The Caucasus is located between the Black and Caspian Seas and is divided into the Pre-Caucasus and Transcaucasia, separated by the Main Caucasian mountain range. There are seven natural foci of plague, geographically located in the Russian Federation, Azerbaijan, Armenia and Georgia. In five of these foci, a highly virulent medieval biovar of the main subspecies of *Yersinia pestis* is common [1]. Three of the five foci–Dagestan plain-piedmont, Terek-Sunzha low-mountain, Central Caucasian high-mountain–belong to the foci of the souslik type and are located in the Pre-Caucasus and the Central Caucasus (S1 Fig). Two more foci belong to the gerbil type foci and are located in Transcaucasia–Transcaucasian plain-piedmont (a group of autonomous plain-piedmont foci: Bozchel, Kobystan, Mil-Karabakh, Jeyranchel and others) and Araks low-mountain foci [2]. In two other foci of the Caucasus, the East Caucasian high-mountain and the Transcaucasian high-mountain (a group of autonomous mountain foci: Gyumri, Sevan and Zangezur-Karabakh), only the сaucasian subspecies–*Y*. *pestis* subspecies *caucasica* (phylogenetic lineage 0.PE2) is currently found, which is virulent mainly for small mouse-like rodents, but can cause isolated cases of plague in humans.

In accordance with genetic nomenclature of branches, the medieval biovar corresponds to the phylogenetic lineage 2.MED [3–6]. The 2.MED lineage is divided into several phylogenetic branches: 2.MED0 (Russia, Central Caucasian high-mountain focus), 2.MED2 and 2.MED3 (China), and 2.MED1 (Eurasian foci). The 2.MED0 strains are endemic to the territory of the Central Caucasian high-mountain focus and are not found in other plague-enzootic regions of the world. The most widespread is the most recent branch 2.MED1, which occupies vast areas of natural plague foci of Eastern Europe and Central Asia. The place of origin of the medieval biovar (lineage 2.MED) is unknown. It is believed that it could have been near the Caspian Sea [7]. In the first half of the 20th century, multiple outbreaks of plague with a high mortality rate occurred in the North-Western and Northern Caspian Sea region with a peak incidence in the 1920s-1930s. In the same period, epizootics among rodents also occurred in this territory. All of these events were preceded by an outbreak of bubonic plague (444 cases, 363 deaths) in the village of Vetlyanka in the Astrakhan province in 1876–1879 [2]. Many dangerous diseases from other countries of the Caspian region were brought into the Russian Empire through the Astrakhan province, located at the confluence of the Volga River into the Caspian Sea. The phylogenetic affiliation of the *Y*. *pestis* strains that caused the outbreak of plague in Vetlyanka is unknown.

A study of *Y*. *pestis* strains obtained in the North-Western and Northern Caspian Sea regions in 1912–1945 showed that the etiological agent of those outbreaks were 2.MED1 strains, as well as strains of another newly identified 2.MED4 branch of the medieval biovar [8]. The 2.MED4 branch existed in the North-Western (Caspian North-Western steppe focus) and Northern (Volga-Ural steppe, Volga-Ural sandy foci) Caspian Sea regions in the first half of the 20th century. In the second half of the 20th century, 2.MED4 strains were no longer isolated. Only one strain of the 2.MED4 branch isolated in the Caucasus is known. It was obtained in 1931 in the Transcaucasian high-mountain focus (Zangezur-Karabakh autonomous focus) from a man who died of pneumonic plague.

In contrast to the North-Western and Northern Caspian Sea regions, there were no large plague outbreaks in the natural foci of the Caucasus during the 20th century, although some local outbreaks or isolated cases of plague did occur. The last major plague outbreaks in the Caucasian natural foci occurred in 1838–1843, and by the end of the 19th century, plague cases in the Caucasian foci were a rare phenomenon. In the 20th century, outbreaks of the plague occurred in the Transcaucasian plain-piedmont focus: in 1914 in the Baku district (30 cases. 30 deaths) and Baku region (50 cases, 50 deaths), in the Nagorno-Karabakh region in 1929–1931 (8 cases, 8 deaths), and in the Araks low-mountain focus in 1948 (15 cases, 12 deaths).

Based on phylogenetic analysis of *Y*. *pestis* strains in combination with epidemiological and epizootiological data, we studied the spatiotemporal patterns of 2.MED1 circulation in the foci of the North-Western and Northern Caspian regions in the 20th and early 21st centuries [8]. We assumed that changes in the level of the Caspian Sea affected the activity of these plague foci. Sharp fluctuations in the level of the Caspian Sea, which had a significant impact on the ecosystem of the Caspian and Turan lowlands, repeatedly occurred in the 20th century under the influence of cyclical climatic changes [9–11]. In our current work, based on a complex of phylogenetic, epidemiological and epizootiological data, we identified the most probable directions of dissemination of the 2.MED1 branch of the medieval biovar in the Caucasus in the 20th-early 21st centuries. This clarification is important for understanding the functioning of natural plague foci and predicting focus activation in the future.

## Materials and methods

### *Yersinia pestis* strains

*Y*. *pestis* strains isolated from natural plague foci of the Caucasus, Northern Caspian region and other foci of Eastern Europe and Central Asia from carriers and vectors of plague, as well as from humans were studied (S1 Table). The strains were obtained from gerbils (*Rhombomys opimus*, *Meriones meridianus*, *Meriones erythrourus*, *Meriones vinogradovi*)– 15, souslik (*Spermophilus pygmaeus*, *Spermophilus fulvus*)– 5, house mouse (*Mus musculus*)– 2, shrew (*Crocidura suaveolens*)– 1, fleas (*Citellophilus tesquorum*, *Ceratophyllus tesquorum*, *Ceratophyllus laeviceps*, *Ctenophillus orienthalis*, *Ctenophtalmus secundus*, *Neopsylla setosa*, *Nosopsyllus laeviceps*, *Ceratophillus caspius*, *Stenoponia insperata*)– 11, and humans– 10.

*Y*. *pestis* was grown in LB broth and LB agar for 24–48 hours. Traditional methods of laboratory diagnostics were used for the assessment of biochemical properties of the strains [12]. Fermentation of glycerol, rhamnose and arabinose was determined on Hiss media (1% peptone water, 0.5% sodium chloride, 1% Andrade indicator, pH 7.2) with the addition of 1% of the corresponding substrates, which were inoculated with 10^8^ CFU of *Y*. *pestis*, and cultivated for 48 hours at 28°C. The reaction was considered positive if the medium became crimson in color. To determine the denitrifying activity, 5 ml of LB broth with 0.1% potassium nitrate were inoculated with 10^8^ CFU of *Y*. *pestis*, cultivated for 72 hours, and then the Griss reagent was added. Crimson staining of the medium indicated the presence of denitrifying activity.

### Whole genome sequencing, SNPs identification, phylogenetic analysis

DNA of *Y*. *pestis* strains was isolated with PureLink Genomic DNA Mini Kit (Invitrogen, USA). Whole genome sequencing of strains was carried out using the Ion GeneStudio S5 System (Thermo Fischer Scientific), according to the manufacturer’s guidelines. Ion Xpress^™^ Plus Fragment Library Kit and Ion Xpress^™^ Barcode Adapter 1–96 Kit were deployed for the primary preparation of samples. The Ion 510^™^ & Ion 520^™^ & Ion 530^™^ Chef Reagents were applied for automated template preparation. For each genome, raw short-read sequences were filtered and quality controlled using the Ion Torrent Suite v5.12.3 software package (https://github.com/iontorrent/TS) and FastQC v0.11.9. (https://github.com/s-andrews/FastQC). The data processing and raw short-read sequences assembling de novo were accomplished with the Ion Torrent Suit software package v5.12.3, Newbler gsAssembler 2.6. [13] and Unicycler v0.4.9. (https://github.com/rrwick/Unicycler). Alignment of the obtained reads to the reference genome of *Y*. *pestis* CO92 (GenBank accession number NC_003143.1) and identification of plasmid sequences was performed using the DNASTAR Lasergene v15.3 software package [14]. Default parameters were used for all software. The sequence reads were assembled into contigs with average coverage per genome being 98.47% (45,9- fold depth) and an average genome assembly size of 4,55 Mb, The average GC% content ranged from 47.39% to 48.06%. Finally, we obtained from 102 to 449 contigs > 1 kb for each genome (S2 Table). The final assemblies were annotated using the NCBI Prokaryotic Genome Annotation Pipeline (PGAP) v6.1 [15]. Each genome contained from 2,445 to 3,978 coding sequences. Core SNPs were identified by aligning contigs of *Y*. *pestis* strains to CO92 genome through Snippy 4.6. software program, then, 28 homoplastic SNPs were excluded. Core SNP calls for all *Y*. *pestis* strains used are shown in S3 Table. The resulting set of SNPs included only the core region of the genome. Using the Snippy software package (https://github.com/tseemann/snippy) provides a set of core SNPs that can be used for constructing high-resolution phylogenetic trees, since it excludes sites of possible recombination. The jModelTest2 program [16] was used to select a model of nucleotide substitutions. Based on the adjusted Akaike criterion (AICc), the GTR model was selected. The Maximum Likelihood tree was constructed using software: SeaView 5.0.4. [17] PhyML-3.1, GTR model and 500 bootstrap replications. The visualization of the dendrogram was carried out in the FigTree 1.4.3 program (http://tree.bio.ed.ac.uk/software/figtree/).

## Results

In this work, we studied 46 strains of *Y*. *pestis* of the medieval biovar isolated in the plague foci of the Caucasus, the Caspian Sea and Aral Sea regions over the period of 1922–2014. The studied strains included strains from the foci of the Caucasus: Central Caucasian high-mountain (2 strains), Terek-Sunzha low-mountain (3), Dagestan plain-piedmont (2), Araks low-mountain (2) foci, as well as from the Zangezur-Karabakh mountain focus (2)–an autonomous focus of the Transcaucasian high-mountain focus, and from three autonomous foci of the Transcaucasian plain-piedmont focus: Bozchel (1), Milsko-Karabakh (1), Jeyranchel (1), Kobystan (8). To compare, strains from the Caspian North-Western steppe (5), Volga-Ural steppe (1), Volga-Ural sandy (5), Ustyurt desert (2), North Aral Desert (2), Mangyshlak desert (2), Aral-Karakum desert (1), Karakum desert (3), and Caspian sandy (3) foci were also used (S1 Table, S2 Fig). An analysis of the biochemical characteristics of the strains revealed that they all have properties typical of the medieval biovar. They do not reduce nitrates or ferment rhamnose, but do consume glycerol and arabinose.

**Fig 1:**
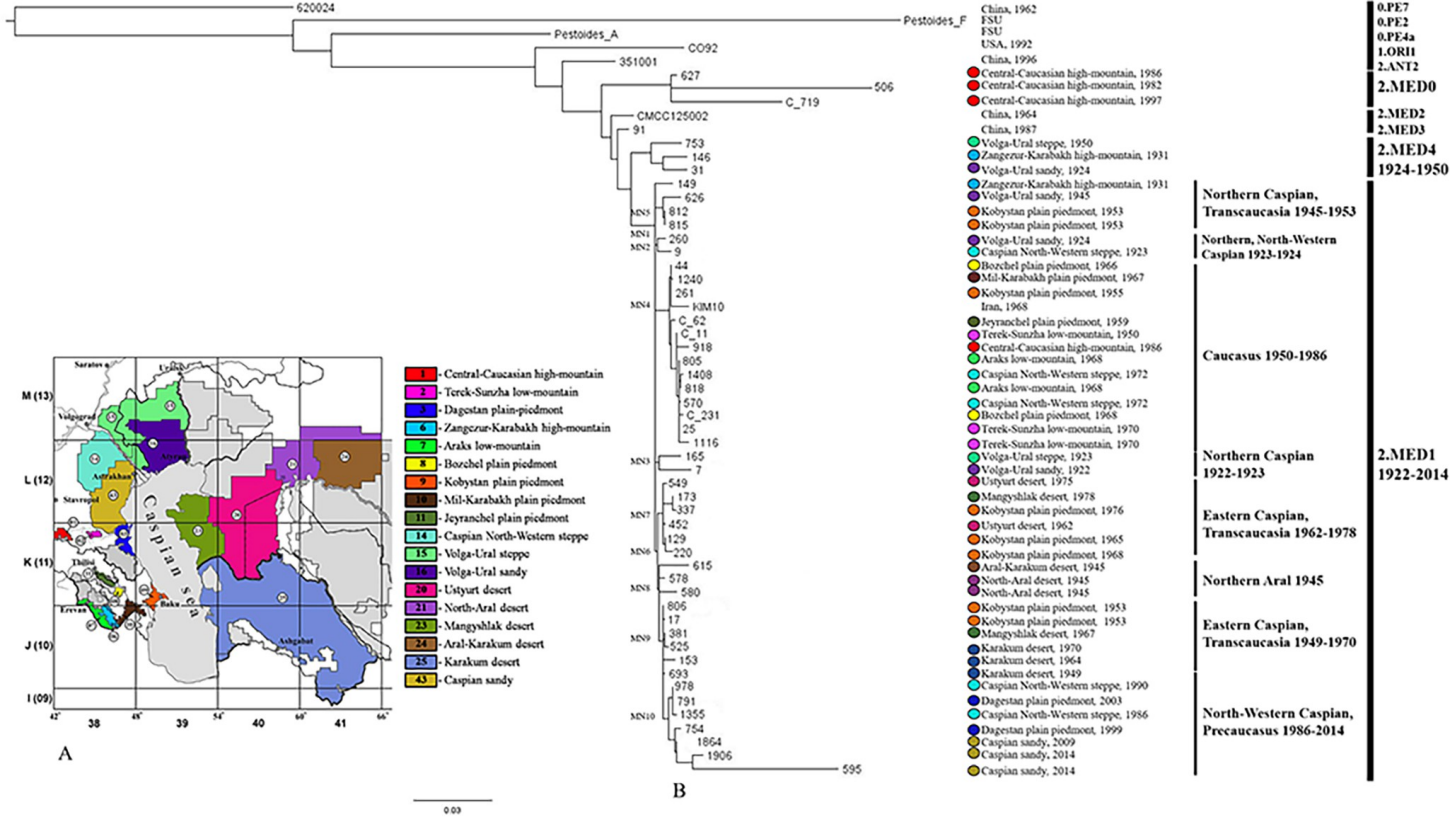
Maximum Likelihood dendrogram of phylogenetic relations of *Yersinia pestis* strains of the medieval biovar of the 2.MED1 phylogenetic branch from the plague foci of the Caucasus, the Northern Caspian and the Northern Aral Sea regions, and other foci of the world. A. Map of natural plague foci of the Caucasus, the Northern Caspian and the Northern Aral Sea regions in the Russian Federation and neighboring countries. The numbers correspond to the classification of plague foci adopted in Russia and other countries of the Commonwealth of Independent States. B. Maximum Likelihood tree based on 1324 core SNPs identified among 25 *Y*. *pestis* strains of the medieval biovar from the Caucasus, as well as among 24 strains of the medieval biovar from the Northern Caspian and Northern Aral Sea regions and 8 strains of different phylogenetic lineages from other regions of the world. SNPs in MN1-MN10 phylogenetic nodes are listed in the S4 Table.

For phylogenetic analysis, whole genome sequences of all 46 strains used were taken. Of these, 18 strains were sequenced by us in this study, and 26 strains were sequenced by us earlier (S1 Table). The nucleotide sequences of another 13 *Y*. *pestis* strains were taken from the NCBI GenBank database. Four of them belonged to the 2.MED1 phylogenetic branch–strains from the Terek-Sunzha low-mountain focus (1), Transcaucasian plain-piedmont focus: Bozchel (1) and Dzheyranchel (1) autonomous foci, and from Kurdistan, Iran (1). Another nine strains from the NCBI GenBank database represented other phylogenetic lineages– 0.PE7, 0.PE2, 0.PE4a, 1.ORI, 2.MED0, 2.MED2, and 2.MED3 (Fig 1, S1 Table).

According to the whole genome SNP analysis, based on 1324 core SNPs, a dendrogram of phylogenetic relations of *Y*. *pestis* strains from the plague foci of the Caucasus was constructed (Fig 1).

In total, whole genome sequences of 25 *Y*. *pestis* strains of the medieval biovar from the foci of the Caucasus and Transcaucasia, 24 strains of the medieval biovar from the Northern Caspian and Northern Aral Sea regions, and 3 strains of this biovar from Iran and China were used to construct the dendrogram. On the dendrogram, *Y*. *pestis* strains of the phylogenetic lineage 2.MED are divided into sequentially diverging branches: 2.MED0, 2.MED3, 2.MED2, 2.MED4, and 2.MED1. The 2.MED1 branch is preceded by the 2.MED4 branch with two strains from the Volga-Ural sandy (1924) and Volga-Ural steppe (1950) foci, and one strain from the Zangezur-Karabakh mountain autonomous focus (1931) included in it. 2.MED4 is followed by 2.MED1, the base trunk of which is represented by a polytomy (MN1 –Medieval Node 1, 12 SNPs in S4 Table), from which sub-branches and clusters outstretch, including *Y*. *pestis* strains from the North-Western, Northern, and Eastern Caspian Sea regions, as well as from the Caucasus and Transcaucasia. This large polytomy indicates a rapid expansion of *Y*. *pestis* across the Caspian Sea region, Caucasus, and Transcaucasia.

The earliest strains on the polytomy are represented by two sub-branches No. 2 (MN2, 1 SNP) from the Northern, North-Western Caspian and No. 3 (MN3, 1 SNP) from the Northern Caspian Sea regions of the early 20th century (1922–1924). All of these strains were isolated from humans and were the etiological agents of plague outbreaks with high mortality rates that occurred in the Caspian North-Western steppe, Volga-Ural steppe and Volga-Ural sandy foci at the beginning of the past century. These early strains departing practically from the trunk of the polytomy (1 SNP), together with a large number of plague cases in this territory, suggest that the first manifestations of the spread of 2.MED1 in the Caspian Sea region and the Caucasus in the early 20th century began in the North-Western and Northern Caspian regions. This assumption is also supported by the data of the Maximum Parsimony dendrogram of phylogenetic relations of *Y*. *pestis* strains of the 2.MED1 phylogenetic branch from the plague foci of the Caucasus, the Northern Caspian and the Northern Aral Sea regions (S3 Fig). On this dendrogram, the strains from the North-Western and Northern Caspian of 1923–1924 precede all other 2.MED1 strains from the regions of the Caspian and Aral Seas and the Caucasus.

The earliest of all studied 2.MED1 strains from the Caucasus and the only clinical strain among them, 149, was isolated in the Zangezur-Karabakh mountain autonomous focus in 1931. It branches directly from the trunk of the 2.MED1 polytomy (5 SNPs). The second, large Caucasian sub-branch of the 2.MED1 branch–No. 4 (MN4, 7 SNPs) consists almost exclusively of strains isolated in the Caucasus and Transcaucasia from 1950–1986. Earlier strains of this sub-branch are absent, and therefore the exact place of its origin is unclear. The strains of sub-branch No. 4 were obtained from carriers and vectors of plague, which indicates the persistence of this sub-branch in the ecosystem of Caucasian foci in the second half of the 20th century. Sub-branch No. 4 is divided on the dendrogram into two clusters, the first of which includes strains from the Mil-Karabakh, Bozchel, and Kobystan autonomous foci of the Transcaucasian plain-piedmont focus and the KIM10 strain from Kurdistan, Iran. The second cluster consists mainly of strains of the Central Caucasus, the Pre-Caucasus and the North-Western Caspian region.

**Fig 2:**
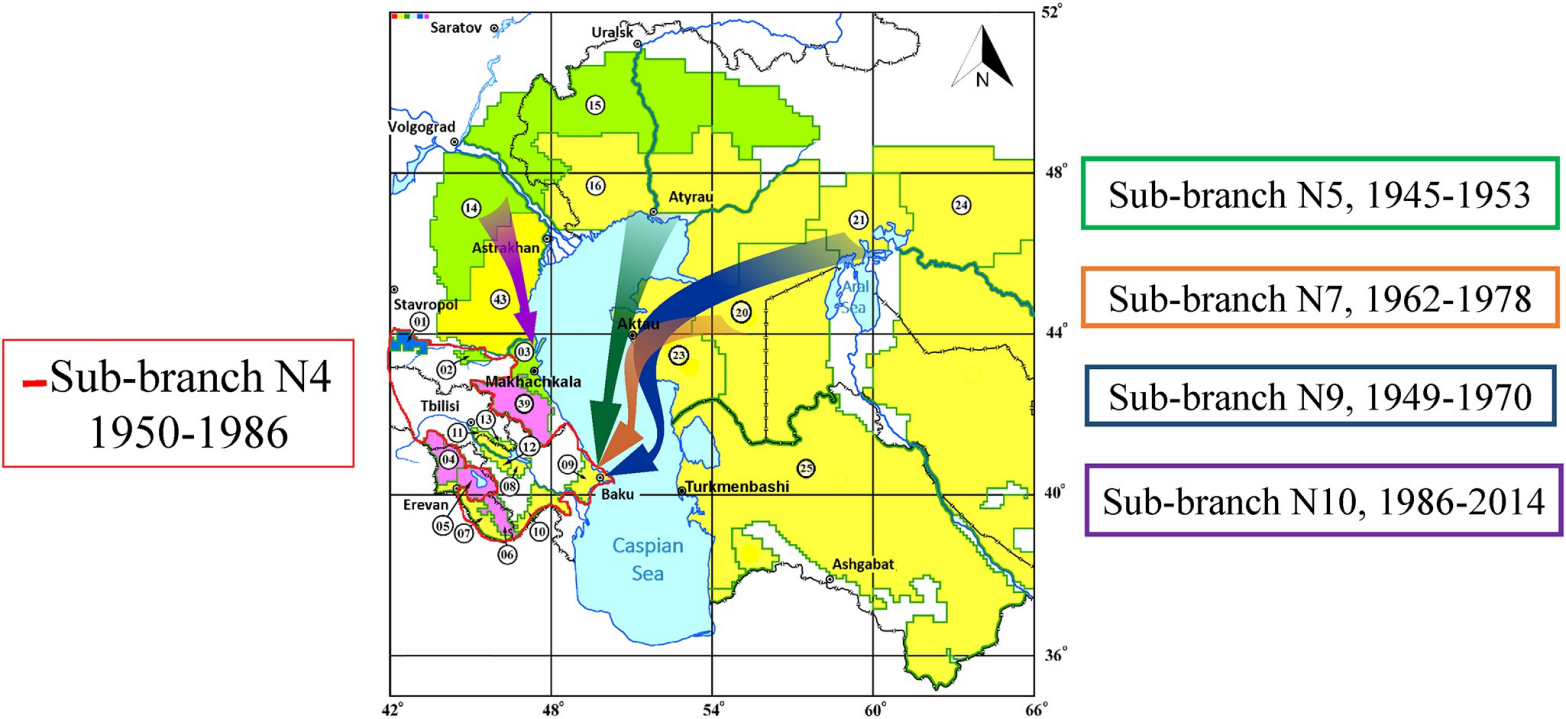
Hypothetical directions of dissemination of the 2.MED1 phylogenetic branch of *Yersinia pestis* of the medieval biovar in the Caucasus in the 20th-21st centuries. Colored arrows indicate the directions of introduction of the 2.MED branch to the Caucasus. The area of distribution of the Caucasian sub-branch No. 4 of 2.MED1 branch in the foci of the Caucasus is circled with a red line. Green color marks plain and low-mountain foci of the souslik type, yellow–plain and low-mountain foci of the gerbil type, blue–high-mountain foci of the souslik type, pink–high-mountain foci of the vole type. The numbers correspond to the classification of plague foci adopted in Russia and other countries of the Commonwealth of Independent States: 1 –Central-Caucasian high-mountain, 2 –Terek-Sunzha low-mountain, 3 –Dagestan plain-piedmont, 4–6 –Transcaucasian high-mountain (a group of autonomous mountain foci: Gyumri, Sevan and Zangezur-Karabakh), 7 –Araks low-mountain, 8–13 Transcaucasian plain piedmont (a group of autonomous foci: Bozchel, Kobystan, Mil-Karabakh, Jeyranchel, Ganja-Kazakh, York), 14 –Caspian North-Western steppe, 15 –Volga-Ural steppe, 16 –Volga-Ural sandy, 20 –Ustyurt desert, 21 –North-Aral desert, 23 –Mangyshlak desert, 24 –Aral-Karakum desert, 25 –Karakum desert, 39 –East Caucasian high-mountain, 43 –Caspian sandy foci.

The remaining strains, isolated from the Caucasus in 1953–2003, did not belong to these Caucasian sub-branches (extending directly from the stem of the 2.MED1 polytomy), but were introduced into the Caucasus from the foci of the Caspian and Northern Aral Sea regions. For example, sub-branch No.5 (MN5, 4 SNP) is composed of three strains, the first of which was isolated in the Volga-Ural sandy focus in 1945 from a human, and two strains of the cluster were obtained in 1953 from fleas in the Kobystan plain-piedmont autonomous focus. Apparently, those strains were introduced into the Kobystan focus from the Northern Caspian region (Fig 2).

Sub-branch No. 6 (MN6, 1 SNP) offshoots from the trunk of the polytomy. It is divided into two sub–branches–the Eastern Caspian (No. 7, MN7, 3 SNPs) and the Northern Aral (No. 8, MN8, 1 SNPs), which demonstrates the further expansion of the 2.MED1 branch to the east. Sub-branch No. 7 of the Eastern Caspian Sea region includes strains that date back to 1962–1978 from the Ustyurt desert, Mangyshlak desert and Kobystan plain-piedmont autonomous foci. This population is the likely source for the introduction of 2.MED1 strains through the Caspian Sea into the Kobystan natural plague focus in 1965–1968. Sub-branch No. 7 includes two separate strains from the Ustyurt desert focus and two additional clusters, one of which consists of strains from the Kobystan focus (1965, 1968), and the second of which consists of strains from the Mangyshlak (1978) and Kobystan (1976) foci. This suggests a path of spread for sub-branch No. 7 from the Ustyurt focus to the Mangyshlak focus and further through the Aktau port of the Mangyshlak Peninsula across the Caspian Sea to the Kobystan autonomous focus (Port Baku, Apsheron Peninsula) in Transcaucasia (Fig 2, S5 Fig).

Sub-branch No. 8 consists of strains from the Northern Aral Sea region from 1945, isolated in the North Aral desert and in the Aral-Karakum desert foci. The ancestors of these strains gave rise to a new polytomy No. 9 (MN9, 2 SNP), which includes strains from 1949–2014 collected from the Eastern Caspian region (Mangyshlak and Karakum desert foci), Transcaucasia and the Pre-Caucasus (Kobystan autonomous and Dagestan plain-piedmont foci), as well as from the North-Western Caspian region (Caspian North-Western steppe, Caspian sandy foci). The presence of this polytomy testifies to the favorable conditions for the spread and introduction of the 2.MED1 branch that developed again in the second half of the 20th century in different territories of the Caspian Sea Region, the Caucasus and Transcaucasia. Strains originating from polytomy No. 9 are descendants of strains from the Northern Aral Sea region of 1945, in the foci of which the 2.MED1 branch survived a long inter-epizootic period in the middle of the 20th century. This inter-epizootic period occurred in the foci of the Northern and North-Western Caspian Sea regions, Caucasus and Transcaucasia against a background of climate warming and a sharp drop in the level of the Caspian Sea.

Directly from the stem of this new polytomy, individual strains from the Karakum desert focus, autonomous Kobystan plain-piedmont focus and a small cluster from the Mangyshlak and Karakum desert foci from 1949–1970 branch off. The most probable path of entry of this sub-branch into Transcaucasia is from the Northern Aral Sea region through the Karakum and Mangyshlak foci to the Kobystan autonomous focus across the Caspian Sea in the late forties-early 1950s. At the same time, the phylogenetic proximity of strains from the Karakum and Kobystan foci cannot exclude the possibility that the local transfer of strains could also have occurred in the opposite direction, from the Kobystan to the Karakum natural foci.

At the end of the 20th century (1978–2014), polytomy No. 9 gave rise to a new sub-branch No. 10 (MN10, 3 SNPs), represented now by strains from the foci of the North-Western Caspian Sea region and the Pre-Caucasus. This sub-branch was likely introduced into the Pre-Caucasus (Dagestan plain-piedmont focus; 1999, 2003) by another way—through the North-Western Caspian Sea region (Caspian North-Western steppe, Caspian sandy foci) (Fig 2).

In general, the phylogenetic analysis of *Y*. *pestis* strains obtained over a period of about a hundred years (1922–2014), together with epidemiological and epizootic data and climatic observations, testifies to intensive processes of dissemination of the 2.MED1 phylogenetic branch in the foci of the North-Western, Northern and Eastern Caspian Sea regions, as well as the Pre-Caucasus and Transcaucasia in the 20th century.

## Discussion

The medieval biovar of the main subspecies of *Y*. *pestis*, phylogenetic lineage 2.MED is widely distributed in Eurasia in steppe, semi-desert, desert, plain-piedmont, low-mountain and high-mountain landscapes with different composition of plague carriers and vectors. The exact place of origin of the 2.MED lineage is not known. The earliest of the branches of the medieval biovar 2.MED0 has been preserved in the Central Caucasian high-mountain focus in Russia. Another, later branch, 2.MED4, existed in the Northern and North-Western Caspian regions at the beginning of the 20th century and possibly even earlier. From the same region, the last branch, 2.MED1, of the medieval biovar began to spread around the Caspian Sea southward to the Caucasus and eastward to the Eastern Caspian and Northern Aral Sea regions. The climate in the 19th century and the beginning of the 20th century in the Caspian lowland was cool with a large amount of precipitation, and the level of the Caspian Sea during this period was consistently high [9–11], http://www.geogr.msu.ru/casp/. This had a positive effect on the ecosystem of natural plague foci of the Caspian region. Against this background, strains of the medieval biovar caused multiple outbreaks of plague in the North-Western and Northern Caspian Sea regions, as well as outbreaks and isolated cases of plague in Central and East Asia in the 20th and 21st centuries [18, 19].

In the Caucasus, infection with plague has been known for a long time. According to historical chronicles, outbreaks and epidemics of plague occurred there throughout the new era and in prehistoric times [20]. The phylogenetic affiliation of *Y*. *pestis* which caused outbreaks of plague in the Caucasus in the 19th century is unknown. In the 20th century, plague infections in the Caucasus occurred in the Transcaucasian plain-piedmont (1910, 1914, 1929), and then in the Araks low-mountain (1948, 1967) and Dagestan plain-piedmont (1951) foci. As a rule, those were small outbreaks or isolated cases of plague. Two outbreaks happened in the Kobystan plain-piedmont autonomous focus in 1914. During one of them, 30 people got sick and died, and during the other 50 people got sick and died of pneumonic plague. Separate cases of plague also took place in the autonomous foci of the Caucasian high-mountain focus: in Zangezur-Karabakh in 1931 and 1975, and in Gyumri in 1958 and 1969 [18]. Two of those cases in 1958 and 1975 were caused by the Caucasian subspecies–*Y*. *pestis* ssp. *caucasica*, but such cases are rare [21].

This study reveals that two cases of plague in the Zangezur-Karabakh mountain autonomous focus in 1931 were caused by 2.MED4 and 2.MED1 strains. It can be assumed that the incidence of plague in the Caucasus at the very beginning of the 20th century (Transcaucasian plain-piedmont focus—1910, 1914, 1929) could also have been caused by 2.MED1 (possibly together with 2.MED4), as we showed for the foci of the North-Western and Northern Caspian regions. Directly from the stem of the 2.MED1 polytomy of the first half of the 20th century, sub-branch No. 4 evolved, which was preserved in the Caucasus through the second half of the century (Fig 1). At the same time, during the middle and in the second half of the 20th century, intensive processes of introduction of 2.MED1 into Transcaucasia and the Pre-Caucasus from the Northern and North-Western Caspian, Eastern Caspian and Northern Aral Sea regions took place. The rate of spread of 2.MED1 may have exceeded even the rate of spread of the phylogenetic lineage 1.ORI, which caused the third plague pandemic [22]. We have indicated that the second wave of 2.MED1 spread likely began in the late 1940s-early 1950s, when the 2.MED1 population preserved in the Northern Aral Sea during the inter-epizootic period gave rise to a new polytomy that swept the North-Western, Northern and Eastern Caspian regions and the Caucasus (Figs 1, 2; S3 and S4 Figs).

Interestingly, strains that came from the Northern Caspian Sea region (sub-branch No. 5), and descendants of strains from the Northern Aral Sea region (sub-branch No. 9) simultaneously circulated in the Kobystan autonomous focus in Transcaucasia in 1953 (Fig 1). In 1955–1966, strains of the local Caucasian sub-branch No. 4 were isolated here, and in 1965–1968 strains that came from the Eastern Caspian Sea region (sub-branch No. 7) were isolated. In a previous publication, we showed that in the period of 1962–1978 in the Eastern Caspian (Ustyurt Plateau) there was a population of the first wave of distribution of 2.MED1 from the Northern Caspian region of the early 20th century [23]. The Kobystan autonomous focus is located on the Apsheron Peninsula alongside the capital of Azerbaijan–Baku. Frequent introductions of plague to this region could have been due to this region’s active economic ties to other regions of the Caspian Sea. Expansion of sub-branch No. 7 in the 1960s and sub-branch No. 9 in the 1950s into the Kobystan focus from the Eastern Caspian regions most likely occurred through the Mangyshlak (port Aktau) and Karakum (port Turkmenbashi) desert foci across the Caspian Sea with commercial and economic cargo (Fig 2, S5 Fig). Previous studies of 580 strains isolated on the Apsheron Peninsula in the middle of the last century showed that they also belong to the medieval biovar of the main subspecies of the plague microbe [24].

The role of the South Caspian Sea region in the spread of 2.MED1 remains unclear. We assume that 2.MED1 was brought to the North-Western and Northern Caspian in the late 19th—early 20th century from Iran, since there was no plague in other nearby regions. Outbreaks of plague in Iran occurred in 1871–1877, 1899, then in 1906–1924 [25]. They may have been caused by 2.MED1. Their introduction across the Caspian Sea into the North-Western Caspian region in the Astrakhan province in 1876 (Vetlyanka station) or later served as the beginning of the spread of 2.MED1 in the foci of the Caspian, Caucasus and Northern Aral in the 20th century. During the period of 1899–1916, 1,758 cases of plague were registered among the populations on the territory of the Right-bank villages of the Volga in the Astrakhan region, with 1,582 of them ending in death [18, 20]. It also cannot be excluded, that outbreaks in Baku and in the Baku region in 1914 were caused by the importation of the plague directly from Iran. Azerbaijan and Iran are connected by close cultural and economic ties, which could be the reason for historical plague penetrations into the Transcaucasia from the south.

The data presented here indicate that in the plain, plain-piedmont and low-mountain natural foci located in the arid landscapes of semi-deserts and deserts of the Caspian and Turan lowlands, under the influence of unfavorable climatic changes, the disappearance of local populations of *Y*. *pestis* may occur. A new activation of the epizootic process may happen due to the introduction of *Y*. *pestis* from other epizootically active areas and its next rooting in historical focal areas. In this work, we confirmed the introduction of *Y*. *pestis* strains of the medieval biovar of the 2.MED1 phylogenetic branch from the Caspian Sea and the Northern Aral Sea region to the Eastern Transcaucasia and Pre-Caucasus in the second half of the last century. The intensity and high rate of the spread of 2.MED1 were apparently caused by active economic and trade activities in the regions of the Caspian Sea and the Caucasus. The import of *Y*. *pestis* as a result of human economic activity have often led to its introduction into the ecosystems of natural focal areas in the presence of favorable climatic conditions. Another possible mechanism of plague dissemination, such as the natural migratory activity of carriers—rodents (small souslik; midday gerbil, crested gerbil, great gerbil and fleas parasitizing on them) could hardly provide such a high rate of spread of the 2.MED1 population of medieval biovar over the vast expanses of Eastern Europe and Central Asia in the 20th and early 21st centuries.

## Supporting information

S1 Table Strains of *Y*. *pestis* used in this study. (XLSX)

S2 Table Basic statistics of genome assemblies. (XLSX)

S3 Table Genomes used for core SNP calling and phylogenetic analysis of *Y*. *pestis* of 2.MED lineage. (XLSX)

S4 Table SNPs, marker for the key nodes of the Maximum Likelihood tree of *Y*. *pestis* strains, used in this study. (XLSX)

S1 Fig Map of natural plague foci of the Caucasus, the Caspian and the Aral Sea regions. Yellow color marks desert and semi-desert foci of the gerbil type, green–semi-desert and steppe foci of the souslik type, blue–high-mountain foci of the souslik type, pink–high-mountain foci of the vole type. The numbers correspond to the classification of plague foci adopted in Russia and other countries of the Commonwealth of Independent States: 1 –Central-Caucasian high-mountain, 2 –Terek-Sunzha low-mountain, 3 –Dagestan plain-piedmont, 4–6 –Transcaucasian high-mountain (a group of autonomous mountain foci: Gyumri, Sevan and Zangezur-Karabakh), 7 –Araks low-mountain, 8–13 Transcaucasian plain piedmont (a group of autonomous foci: Bozchel, Kobystan, Mil-Karabakh, Jeyranchel, Ganja-Kazakh, York), 14 –Caspian North-Western steppe, 15 –Volga-Ural steppe, 16 –Volga-Ural sandy, 20 –Ustyurt desert, 21 –North-Aral desert, 23 –Mangyshlak desert, 24 –Aral-Karakum desert, 25– Karakum desert, 39 –East Caucasian high-mountain, 43 –Caspian sandy foci.
(TIF)

S2 Fig Places of isolation of *Y*. *pestis* strains on the map of plague foci of the Caucasus, the Caspian Sea and the Northern Aral Sea region. Red circles mark the places of isolation of *Y*. *pestis* strains. Yellow color marks desert and semi-desert foci of the gerbil type, green–semi-desert and steppe foci of the souslik type, blue–high-mountain foci of the souslik type, pink–high-mountain foci of the vole type. The numbers correspond to the classification of plague foci adopted in Russia and other countries of the Commonwealth of Independent States: 1 –Central-Caucasian high-mountain, 2 –Terek-Sunzha low-mountain, 3 –Dagestan plain-piedmont, 4–6 –Transcaucasian high-mountain (a group of autonomous mountain foci: Gyumri, Sevan and Zangezur-Karabakh), 7 –Araks low-mountain, 8–13 Transcaucasian plain piedmont (a group of autonomous foci: Boz-chel, Kobystan, Mil-Karabakh, Jeyranchel, Ganja-Kazakh, York), 14 –Caspian North-Western steppe, 15 –Volga-Ural steppe, 16 –Volga-Ural sandy, 20 –Ustyurt desert, 21 –North-Aral desert, 23 –Mangyshlak desert, 24 –Aral-Karakum desert, 25– Karakum desert, 39 –East Cau-casian high-mountain, 43 –Caspian sandy foci.
(TIF)

S3 Fig Maximum Parsimony dendrogram of phylogenetic relations of *Yersinia pestis* strains of the medieval biovar of the 2.MED1 phylogenetic branch from the plague foci of the Caucasus, the Northern Caspian and the Northern Aral Sea regions, other foci of the world. Maximum Parsimony tree based on 1324 core SNPs identified among 25 *Y*. *pestis* strains of the medieval biovar from the Caucasus, as well as among 24 strains of the medieval biovar from the Northern Caspian and Northern Aral Sea regions and 8 strains of different phylogenetic lineages from other regions of the world. Constructed in MEGA X.
(TIF)

S4 Fig Expanded Maximum Likelihood dendrogram of phylogenetic relations of *Y*. *pestis* strains of the medieval biovar of the 2.MED1 phylogenetic branch from the plague foci of the Caucasus, the Northern Caspian and the Northern Aral Sea regions with the addition of strains at key nodes in comparison with Fig 1. Designation of foci on the map (A) and key phylogenetic nodes on the dendrogram (B) is the same as in Fig 1.
(TIF)

S5 Fig Map of seaports and roads in the Caspian and Caucasus regions. © https://kakdobratsyado.ru/country/kazahstan/goroda-kazahstana/aktau-kazahstan.
(TIF)

## References

[1] Kutyrev VV, Eroshenko GA, Motin VL, Nosov NY, Krasnov JM, Kukleva LM, et al. Phy-logeny and classification of *Yersinia pestis* through the lens of strains from the plague foci of Commonwealth of Independent States. Front Microbiol. 2018; 9:1106. Epub. 2018/5/25. doi: 10.3389/fmicb.2018.01106 29887859 PMC5980970

[2] Onishchenko GG, Kutyrev VV, editors. [Natural Plague Foci in the Territory of Caucasus, Caspian Sea Region, Central Asia and Siberia]. Moscow: “Medicine”; 2004. 192 p. https://www.elibrary.ru/item.asp?id=19525294.

[3] Achtman M, Zurth K, Morelli G, Torrea G, Guiyoule A, Carniel E. *Yersinia pestis*, the cause of plague, is a recently emerged clone of *Yersinia pseudotuberculosis*. Proc Natl Acad Sci U S A. 1999; 96:14043–14048. doi: 10.1073/pnas.96.24.14043 10570195 PMC24187

[4] Morelli G, Song Y, Mazzoni CJ, Eppinger M, Roumagnac P, Wagner DM, et al. *Yersinia pestis* genome sequencing identifies patterns of global phylogenetic diversity. Nat Genet. 2010; 42(12):1140–1143. Epub 2010/11/03. doi: 10.1038/ng.705 . 21037571 PMC2999892

[5] Cui Y, Yu C, Yan Y, Li D, Li Y, Jombart T, et al. Historical variations in mutation rate in an epidemic pathogen, *Yersinia pestis*. Proc Natl Acad Sci U S A. 2013; 110(2):577–582. Epub 2012/12/29. doi: 10.1073/pnas.1205750110 . 23271803 PMC3545753

[6] Demeure CE, Dussurget O, Mas Fiol G, Le Guern AS, Savin C, Pizarro-Cerdá J. *Yersinia pes-tis* and plague: an updated view on evolution, virulence determinants, immune subversion, vac-cination, and diagnostics. Genes Immun. 2019; 20(5):357–370. Epub 2019 Apr 3. doi: 10.1038/s41435-019-0065-0 . 30940874 PMC6760536

[7] Benedictow, OJ. (2004). The Black Death, 1346–1353: the complete history (Boydell & Brewer).

[8] Eroshenko GA, Popov NV, Al’khova ZV, Kukleva LM, Balykova AN, Chervyakova NS, et al. Evolution and circulation of *Yersinia pestis* in the Northern Caspian and Northern Aral Sea re-gions in the 20th-21st centuries. PLoS One. 2021; 16(2):e0244615. eCollection 2021. doi: 10.1371/journal.pone.0244615 . 33571993 PMC7878065

[9] Popov NV, Udovikov AI, Eroshenko GA, Karavaeva TB, Yakovlev SA, Porshakov AM, et al. Impact of the Caspian Sea level fluctuations on the epizootic activity of the Caspian sandy natu-ral plague focus. Med Parazitol (Mosk). 2016; Jan-Mar; (1):12–17. . 27029140

[10] Kasimov NS, Gennadiev AN, Kasatenkova MS, Lychagin MY, Kroonenberg SB, Koltermann P. Geochemical changes in the Caspian salt marshes due to the sea level fluctuations. Earth Science Research. 2012; 1(2):262–278. doi: http%3A//dx.doi.org/10.5539/esr.v1n2p262

[11] Chen J, Tapley BD, Wilson SR, Kostianoy AG. Long-term Caspian Sea level change. Ge-ophysical Research Letters. 2017; June.

[12] Onishchenko GG, Kutyrev VV, editors. [Laboratory Diagnostics of Particularly Dangerous Infectious Diseases. Practice Guidelines]. Moscow: CJSC “Shiko”; 2013. 560 p. https://www.elibrary.ru/item.asp?id=21715269.

[13] Life Sciences Corp. 2010. Part COS De Novo Assembler, GS Reference Mapper, SFF Tools, 454. In 454 sequencing system software manual, v 2.5.3. Life Sciences Corp., Branford, CT.

[14] Burland TG. DNASTAR’s Lasergene sequence analysis software. Methods Mol Biol. 2000; 132:71–91. doi: 10.1385/1-59259-192-2:71 . 10547832

[15] Tatusova T, DiCuccio M, Badretdin A, Chetvernin V, Nawrocki EP, Zaslavsky L, et al., NCBI Prokaryotic Genome Annotation Pipeline. Nucleic Acids Res. 2016; 44:6614–6624. Epub 2016 Jun 24. doi: 10.1093/nar/gkw569 27342282 PMC5001611

[16] Darriba D, Taboada GL, Doallo R, Posada D. jModelTest 2: more models, new heuristics and parallel computing. Nat Methods. 2012; 9(8):772. doi: 10.1038/nmeth.2109 . 22847109 PMC4594756

[17] Gouy M, Guindon S, Gascuel O. SeaView version 4: A multiplatform graphical user interface for sequence alignment and phylogenetic tree building. Mol Biol Evol. 2010; 27(2):221–224. Epub 2009 Oct 23. doi: 10.1093/molbev/msp259 . 19854763

[18] Kutyrev VV, Popova AYu, editors. [Cadastre of Epidemic and Epizootic Plague Manifestations in the Territory of the Russian Federation and Former Soviet Union Countries (1876–2016)]. Saratov: LLC “Amirit”; 2016. 248 p. https://www.elibrary.ru/item.asp?id=29079789.

[19] Li J, Wang Y, Liu F, Shen X, Wang Y, Fan M, et al. Genetic source tracking of human plague cases in Inner Mongolia-Beijing, PLoS Negl Trop Dis. 2019; 15(8):e0009558. eCollection 2021 Aug. doi: 10.1371/journal.pntd.0009558 . 34343197 PMC8362994

[20] Supotnitsy MV, Supotnitskaya NS. Essays on the history of the plague. Book 1. Essay XVIII. The last major plague epidemics in the Caucasus (1838–1843). [Cited 2022 December 19]. https://royallib.com/book/supotnitskiy_mihail/ocherki_istorii_chumi_kniga_i.html

[21] Nikiforov KA, Al’khova ZhV, Kukleva LM, Naryshkina EA, Oglodin EG, Eroshenko GA, et al. Phylogenetic analysis of *Yersinia pestis* strains of the Caucasian subspecies from the foci of the Caucasus and Transcaucasia according to the whole genome sequencing data. Russian Journal of Genetics. 2019; 55(4):426–432. doi: 10.1134/S102279541904007

[22] Xu L, Stige LC, Leirs H, Neerinckx S, Gage KL, Ruifu Y, et al. Historical and genomic data reveal the influencing factors on global transmission velocity of plague during the Third Pan-demic. Proc Natl Acad Sci U S A. 2019; 116(24):11833–11838. doi: 10.1073/pnas.1901366116 31138696 PMC6584904

[23] Eroshenko GA, Kukleva LM, Al’khova ZhV, Balykova AN, Popov NV, Krasnov YaM, et al. Phylogenetic history of Kara Kum desert focus. Problems of Particularly Dan-gerous Infections. 2020; 3:56–61. doi: 10.21055/0370-1069-2020-3-56-61

[24] Akhundov MG, Babenyshev VP, Bocharnikov ON, Isaeva EV, Karpusheva V, et al. Charac-teristics of the course of plague epizootics in Azerbaijan in 1953–1958. The experience of combating them and the prospects of eliminating the natural focus. Proceedings of the Armenian Anti-Plague Station. Yerevan. 1960; 1:79–101.

[25] Shahraki AH, Carniel E, Mostafavi E. Plague in Iran: its history and current status. Epidemiology and Health. 2016; 38: e2016033, doi: 10.4178/epih.e2016033 27457063 PMC5037359

