# Supplementary figures and images for: Retrospective analysis of dissemination of the 2.MED1 phylogenetic branch of Yersinia pestis in the Caucasus
Source: PLoS One. 2023 Mar 29;18(3):e0283670. doi: 10.1371/journal.pone.0283670 (PMC10057742; doi:10.1371/journal.pone.0283670)

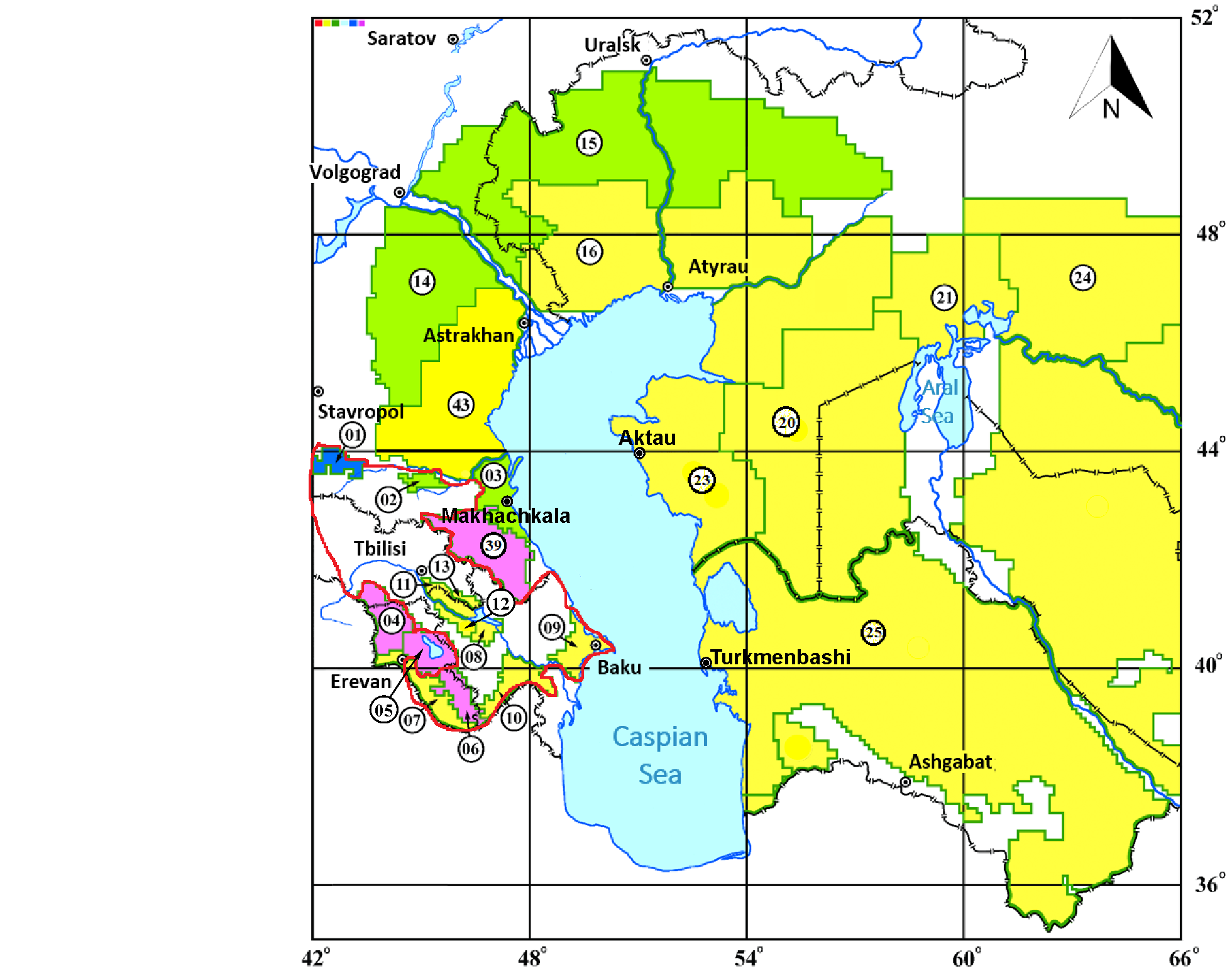

Supplement: S1 Fig — Yellow color marks desert and semi-desert foci of the gerbil type, green–semi-desert and steppe foci of the souslik type, blue–high-mountain foci of the souslik type, pink–high-mountain foci of the vole type. The numbers correspond to the classification of plague foci adopted in Russia and other countries of the Commonwealth of Independent States: 1 –Central-Caucasian high-mountain, 2 –Terek-Sunzha low-mountain, 3 –Dagestan plain-piedmont, 4–6 –Transcaucasian high-mountain (a group of autonomous mountain foci: Gyumri, Sevan and Zangezur-Karabakh), 7 –Araks low-mountain, 8–13 Transcaucasian plain piedmont (a group of autonomous foci: Bozchel, Kobystan, Mil-Karabakh, Jeyranchel, Ganja-Kazakh, York), 14 –Caspian North-Western steppe, 15 –Volga-Ural steppe, 16 –Volga-Ural sandy, 20 –Ustyurt desert, 21 –North-Aral desert, 23 –Mangyshlak desert, 24 –Aral-Karakum desert, 25– Karakum desert, 39 –East Caucasian high-mountain, 43 –Caspian sandy foci. (TIF) [file pone.0283670.s005.tif]

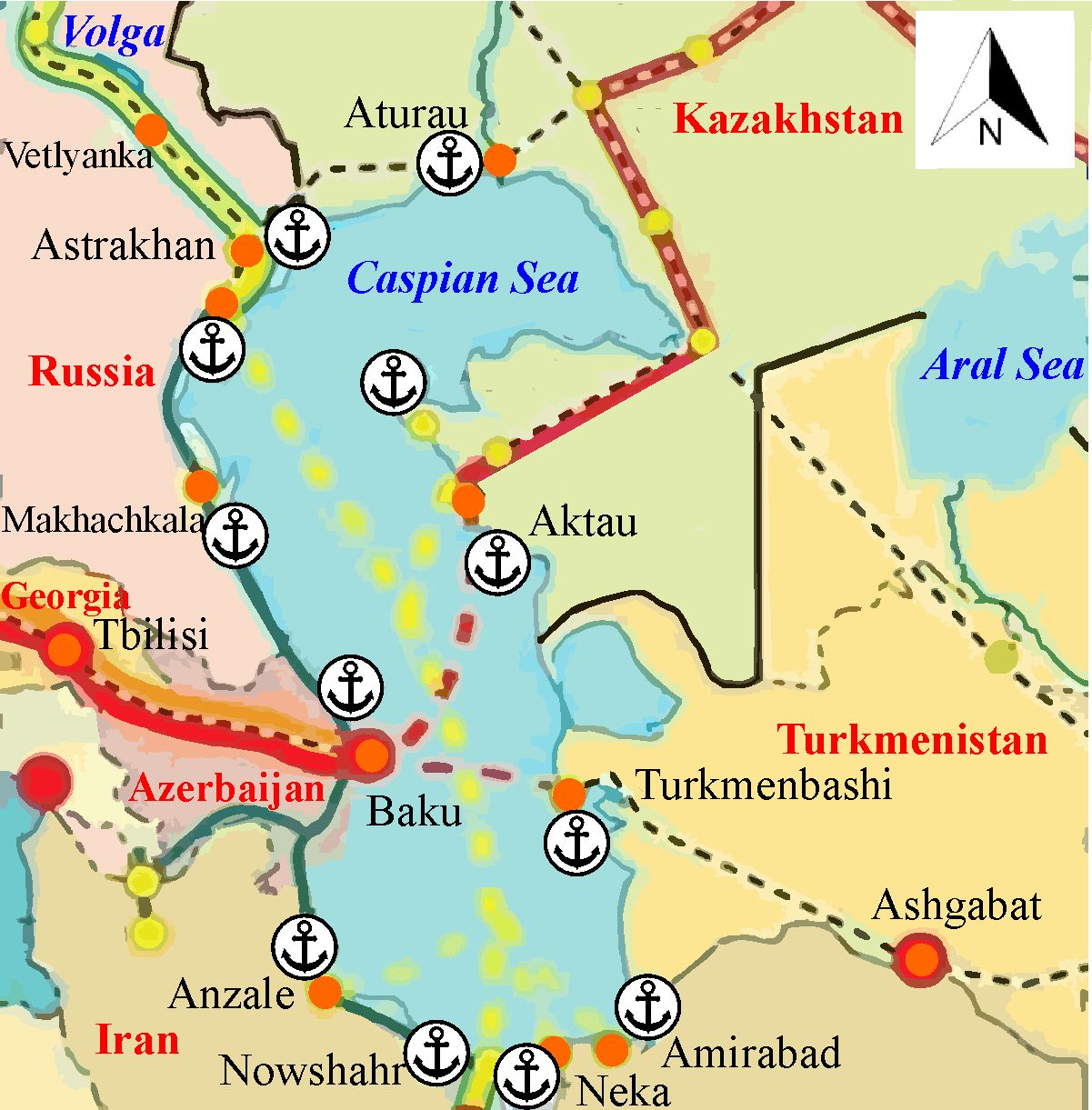

Supplement: S5 Fig — © https://kakdobratsyado.ru/country/kazahstan/goroda-kazahstana/aktau-kazahstan. (TIF) [file pone.0283670.s009.tif]
